# Respiratory Rate Sensing for a Non-Stationary Human Assisted by Motion Detection †

**DOI:** 10.3390/s25072267

**Published:** 2025-04-03

**Authors:** Hsi-Chou Hsu, Wei-Hsin Chen, Yi-Wen Lin, Yung-Fa Huang

**Affiliations:** 1 Department of Computer and Communication, National Pingtung University, Pingtung 91201, Taiwan; chrishsu@mail.nptu.edu.tw; 2 Department of Computer Science and Information Engineering, National Pingtung University, Pingtung 91201, Taiwan; bbb111014@stmail.nptu.edu.tw (W.-H.C.); lwen20460@gmail.com (Y.-W.L.); 3 Department of Information and Communication Engineering, Chaoyang University of Technology, Taichung 413310, Taiwan

**Keywords:** non-stationary, respiratory rate, radar signal processing, ultra-wideband (UWB) radar

## Abstract

Non-contact human respiration rate monitoring can be used for sleep apnea detection and home care. Typically, the human body does not remain stationary for long periods, and body movement can significantly affect the performance of non-contact respiratory monitoring. Because the breathing rate generally remains stable over short periods, using measurements from only a portion of the radar echo signals does not result in significant errors, and these errors will be smaller than those caused by body movement. However, selecting a window size that is too short reduces frequency resolution, leading to increased estimation errors. Choosing an appropriate window length can improve estimation accuracy. In this paper, we propose an algorithm to determine whether the subject is stationary and select the received signal with minimal body movement. Experimental results are compared using alternative schemes, including fast Fourier transform (FFT), short-time Fourier transform (STFT), and RGB-D camera-assisted methods, in terms of root mean square error (RMSE) performance.

## 1. Introduction

Respiration and heartbeat are two key characteristics of vital signs. The cyclic rise and fall of the chest leads to slight changes in the phase and wavelength of electromagnetic waves. By utilizing the echo of wireless signals, these physiological signals can be detected in a non-contact manner, enabling the calculation of breathing rate and heart rate. Common devices used for detecting vital signs include electrocardiograms, smartwatches, and belt sensors. While these devices provide real-time information, they may cause discomfort in the subjects as they are contact-based. Moreover, certain measurement methods, such as electrode patches, may infringe on the subject’s privacy. To address the limitations of contact-based devices, there has been active research into non-contact respiration and heartbeat monitoring methods. The most commonly used non-contact methods include frequency-modulated continuous-wave (FMCW) radar [1,2,3,4,5] and ultra-wideband (UWB) radar [6,7,8,9,10,11]. FMCW radar is a reliable, robust, and harmless tool for continuous monitoring of cardiac and respiratory rates, especially for multiple human targets. It holds great potential for applications in wards and home healthcare [5]. Due to its low power radiation, UWB radar is suitable for long-term monitoring [6]. Currently, UWB radar products such as the XeThru X4M200 developed by Novelda can accurately measure the breathing rate of stationary subjects [12,13]. However, it still cannot measure the breathing rate of non-stationary subjects. When an object or body part moves, it causes a frequency shift (Doppler shift) in radar waves that reflect off it. Micro-Doppler refers to the small frequency shifts caused by subtle movements such as chest expansion and contraction during breathing. These signatures can be analyzed to extract useful information, such as respiratory rate [13].

The Doppler effect is a phenomenon in which the frequency of a wave shifts due to the relative motion between the wave source and the observer. In radar systems, when a target moves toward or away from the radar, the frequency of the reflected signal shifts according to the target’s radial velocity, a phenomenon known as Doppler shift. By measuring this frequency shift, the target’s radial velocity can be estimated. The micro-Doppler effect [14,15] further describes the frequency modulation caused by small-scale movements of an object, such as vibrations, rotations, or limb motions. These subtle movements introduce additional sidebands in the Doppler spectrum, forming what is known as micro-Doppler signatures. One important application of micro-Doppler technology is the remote monitoring of human respiration [16]. When a subject remains stationary, the periodic micro-displacement of the chest due to breathing generates signal peaks in the frequency domain corresponding to the breathing rate, allowing for respiration rate measurement at a certain distance. However, real-world environments are often non-static, and several factors can introduce additional Doppler echoes that obscure the weak breathing signal.

Self-injection locked (SIL) radar has gained popularity due to its low system complexity, excellent sensitivity, and immunity to clutter signals [17]. For stationary subjects, SIL technology allows for rapid extraction of Doppler frequency shift signals from the echo signals, enabling the calculation of respiratory and heartbeat frequencies [18,19,20,21]. For targets with constant velocity translation, the Doppler shift is a time-invariant function. However, if the target experiences vibration or rotation, the Doppler shift caused by these movements becomes time-varying, leading to periodic modulation of the carrier frequency [5]. Therefore, non-contact vital sign sensing for non-stationary subjects remains a challenge. Using self-injection-locked radar with two antennas [20] can mitigate the nonlinear effects of large Doppler phase shifts on the cancellation of body motion artifacts, enabling the extraction of accurate vital signs from the received Doppler-shifted signal. However, the subject must be positioned between the two antennas, which limits usability and convenience.

Time-frequency analysis (TFA) methods, such as short-time Fourier transform (STFT) [22,23] and wavelet transform (WT) [24,25], have been proposed to address this challenge in measuring human vital signs. Wavelet transform offers better frequency resolution at low frequencies, which makes it perform better than STFT. However, the computational complexity of wavelet transform is relatively high. In [22], an FFT-based spectrum averaging procedure was implemented using STFT with non-overlapping windows. Some windows may not show clear peaks due to noise and random body motion. To improve accuracy, Giordano and Islamoglu [23] proposed considering only the three peaks with the highest magnitudes. Additionally, the STFT divides a long time sequence into shorter segments and computes the FFT separately by sliding an analysis window over the signal. Due to the limited length of temporal signals, spectral leakage occurs in the signal spectrum. When the breathing signal is corrupted by random movement, the energy at the true frequency may be low. Spectral leakage can significantly affect estimation performance. Traditionally, spectral leakage can be reduced by using window functions such as Hamming and Kaiser [26]. For example, the Hamming window provides good frequency resolution and moderate spectral leakage [27]. Experimental studies in [28] show that the most efficient performance is achieved using the Hamming and Kaiser window functions.

A major drawback of the TFA scheme is its high computational complexity. To improve vital signal measurement for non-stationary subjects, Khan and Cho [9] used the Novelda NVA6201 radar transceiver and proposed utilizing the width of the autocorrelation signal of the received signal to distinguish whether the subject is stationary or moving. If human movement is detected, the current measurement is ignored, and the previous sample is retained until the subject becomes stationary. Essentially, when human motion is detected, signal processing techniques can be applied to mitigate the impact of body movement. Many studies have proposed methods to detect human movement [29,30,31,32,33]. In our previous work [34], an acceleration-based method [31] was used to identify received signal samples taken when subjects were non-stationary. This paper is an extension of our previous work. In this paper, we propose several improvements, such as directly selecting the window with the smallest accumulated acceleration factor to eliminate the issue of threshold value selection and adaptively adjusting the window length to achieve better estimation performance. Additionally, we conduct detailed verification through various test cases and compare the performance with STFT and RGB-Depth (RGB-D) camera-assisted schemes.

The rest of this paper is organized as follows. Section 2 describes the signal model. Section 3 presents the proposed algorithm. Section 4 provides experimental results of the proposed scheme, comparing them with FFT, STFT, and RGB-D camera-assisted schemes. Finally, Section 5 summarizes and concludes the paper.

## 2. Signal Model

**Figure 1:**
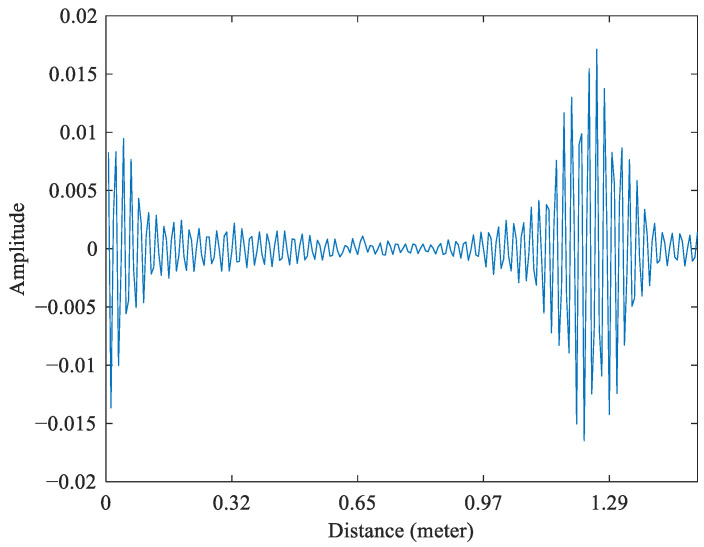
The echo signal received through the X4M200.

This paper uses ultra-wideband (UWB) technology to measure respiratory rates. UWB is a low-power, high-speed wireless network communication technology and a non-carrier communication technology. It does not use a continuous sine wave but transmits information through narrow pulses from nanoseconds to picoseconds. The signal emitted by the X4M200 module is the frequency-shifted Gaussian pulse, which makes a good candidate for an UWB carrier due to relatively good spectrum filling, short duration in time, and ease of implementation in CMOS. A frequency-shifted Gaussian pulse is expressed by [8].

(1) $$s(t)=p(t)\cos\left(\omega_{c}t\right)=V_{t}exp\left(-\frac{t^{2}}{2\beta^{2}}\right)\cos\left(\omega_{c}t\right),$$

where p(t) is the envelope of the Gaussian pulse, $V_{t}$ is the pulse amplitude, $\omega_{c}$ is the center frequency, $\beta ={\left(2\pi B_{w}{\left({log}_{10}\left(e\right)\right)}^{1/2}\right)}^{-1}$, and $B_{w}$ is the bandwidth. The radar captures reflections of every object in its field of view. The amount of energy reflected in the radar is referred to as the object’s radar cross section (RCS). In addition to the original reflection from an object, there are also multipath reflections for an object at distances further away than the object. Figure 1 shows the echo signal received through the X4M200. The strong pulse starting at bin 0 is caused by energy going directly from the transmitting antenna to the receiving antenna, referred to as the direct path. The amount of energy in the direct path is determined by the antenna isolation and will differ for different modules and antenna setups.

The *n*-th transmitted UWB signal can be expressed as

(2) $$s(t-nT)=p(t-nT)\cos\left(\omega_{c}t-n\omega_{c}T\right),$$

where T is the time duration between successive pulses. This is true, assuming the displacement of breathing is a simple harmonic motion and the respiration amplitude $d_{b}$ is a constant. Thus, the time delay caused by the displacement of breathing is given as

(3) $$b\left(t\right)=t_{b}\sin\left(2\pi f_{b}t+\theta \right),$$

where $f_{b}$ is the frequency of respiration, θ is the initial phase, $t_{b}={2d}_{b}/c$ is the round-trip propagation time for the $d_{b}$, and *c* is the light speed. The echo signal reflected from the human body is given as

(4) $$r(t-nT)=s(t-nT-t_{d}-b(t))+N\left(t-nT\right),$$

where $t_{d}$ is the time delay of the transmission path between the UWB radar and the subject, and N(t) is the additive Gaussian noise. The baseband signal is obtained by

(5) $$\begin{array}{l} x(t-nT)=r(t-nT)e^{j\omega_{c}t}.\\ =\frac{1}{2}p(t-nT-t_{d}-b(t))\left\{e^{j\left[\omega_{c}t-n\omega_{c}T-{\omega_{c}t}_{d}-\omega_{c}b(t)\right]}+e^{-j\left[\omega_{c}t-n\omega_{c}T-{\omega_{c}t}_{d}-\omega_{c}b(t)\right]}\right\}e^{j\omega_{c}t}+N\left(t-nT\right)e^{j\omega_{c}t}\\ =\frac{1}{2}p(t-nT-t_{d}-b\left(t\right))\left\{e^{2j\omega_{c}t-j\omega_{c}\left[nT+t_{d}+b\left(t\right)\right]}+e^{j\omega_{c}\left[nT+t_{d}+b\left(t\right)\right]}\right\}+N\left(t-nT\right)e^{j\omega_{c}t}. \end{array}$$

The double frequency components, $e^{2j\omega_{c}t-j\omega_{c}\left[nT+b\left(t\right)+\tau_{l}\right]}$ are filtered through a low-pass filter (LPF). Thus, the baseband signal is given by

(6) $$x\left(t-nT\right)=\frac{1}{2}p\left(t-nT-t_{d}-b\left(t\right)\right)e^{j\omega_{c}\left[nT+t_{d}+b\left(t\right)\right]}+N\left(t-nT\right)e^{j\omega_{c}t}.$$

Assuming there are $N_{s}$ received frames, the aggregated baseband signal is therefore expressed as

(7) $$\begin{array}{ll} y(t) & =\sum_{n=1}^{N_{s}}x(t-nT)\\ & =\frac{1}{2}\sum_{n=1}^{N_{s}}\left\{\frac{1}{2}p\left(t-nT-t_{d}-b\left(t\right)\right)e^{j\omega_{c}\left[nT+t_{d}+b\left(t\right)\right]}+N\left(t-nT\right)e^{j\omega_{c}t}\right\}. \end{array}$$

The aim of respiration rate detection is to estimate the $f_{b}$ term in Equation (3). The FFT is a popular algorithm used to obtain frequency domain representation of input data and obtain the respiration frequencies. When a human body is stationary, that is, when $t_{d}$ is almost invariant, the $f_{b}$ can be obtained accurately by performing the FFT on $y\left(t\right)$. Otherwise, in non-stationary situations, because $t_{d}$ changes rapidly, it will cause an estimation error of $f_{b}$.

## 3. Proposed Algorithm

The experimental device uses the X4M200 module, a sensor based on UWB radar technology that uses discontinuous pulse signals for measurement. The measurement is carried out in a low-interference environment, and the distance between the subject and the X4M200 is 1 m. During the measurement process, the subject made random movements.

**Figure 2:**
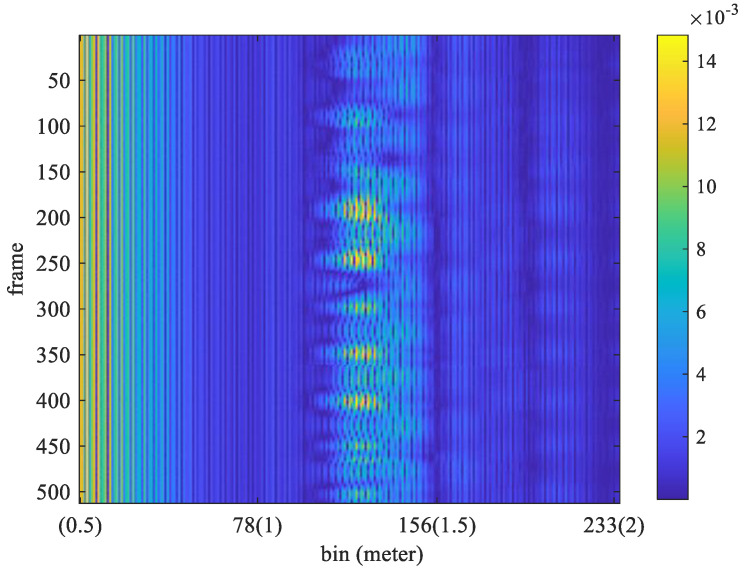
Received frames for stationary condition.

Table 1 illustrates the experimental conditions. The frame rate is 16 frames per second and the sampling interval is $T_{f}=1/16$ s. Therefore, there were 512 sample frames in the time interval of 32 s. The sampling frequency is 23.328 GHz, then the sampling interval, $T_{∆}$, is equal to 0.042867 *ns.* Therefore, the interval for each bin is equal to $\left(T_{∆}\times c\right)/2=0$.0064 m. Therefore, the interval for each bin is given by ($T_{∆}$ × *c*)/2 = 0.0064 m, where *c* denotes the speed of light. Set the measurement range to be from 0.5 to 2 m, and there will be 235 fast-time samples within this range. Let the sample of *y*(*t*) sampled at *t = n*$T_{f}+mT_{∆}$ be denoted as $y\left[n,m\right]$, where *n* satisfies 1≤n≤N, and *m* satisfies 1≤m≤M. Here, *N* represents the total number of frames in a received block, and *M* denotes the number of samples per frame. For example, the received signal under a stationary condition is shown in Figure 2, where *N* equals 512 and *M* equals 235. The FFT output for *m*-th column (bin) of the sample matrix shown in Figure 2 can be computed by

(8) $$Y_{m}\left[k\right]=\sum_{n=1}^{N}y[n,m]e^{-j2\pi k(n-1)/N}.$$

**Figure 3:**
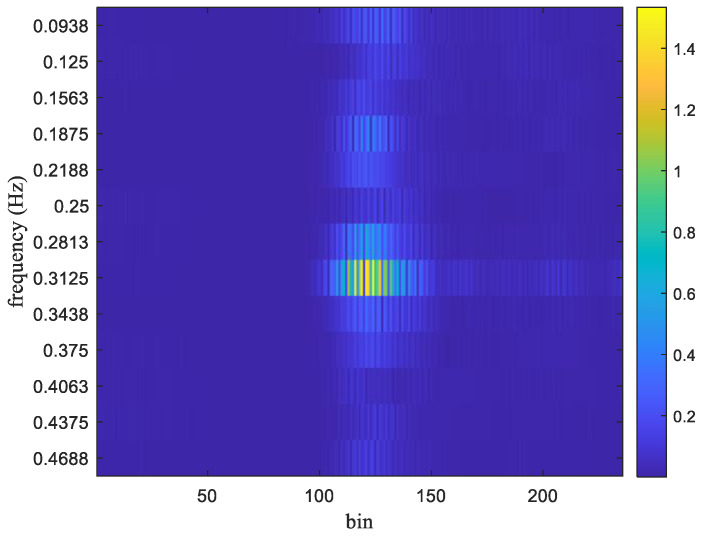
The FFT output of the received frames shown in Figure 2.

**Figure 4:**
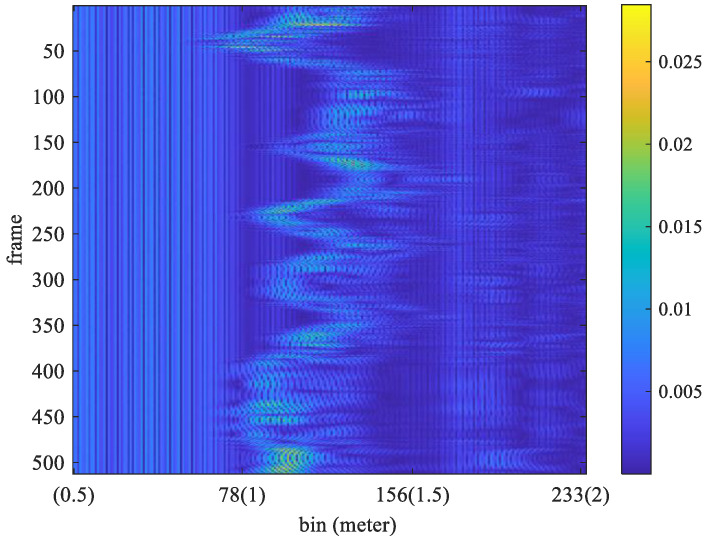
Received frames for non-stationary conditions.

**Figure 5:**
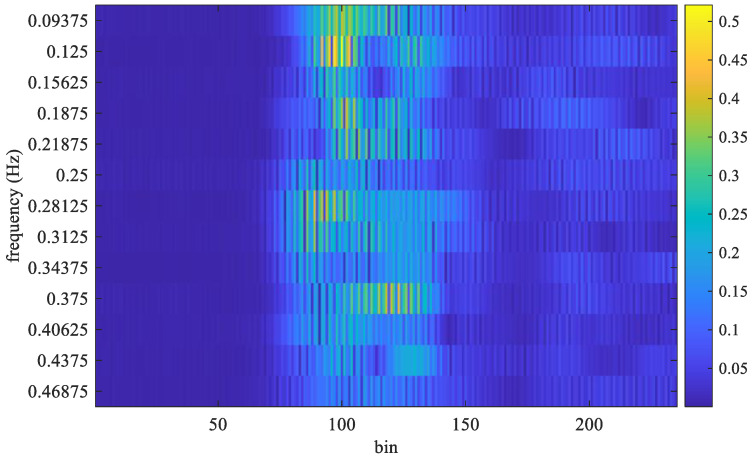
The FFT output of the received frames shown in Figure 4.

The FFT output for the frames received in Figure 2 is shown in Figure 3. The respiration frequency is determined by selecting the frequency with the highest magnitude. Thus, it can select a couple of *m* and *k* that make $Y_{m}\left[k\right]$ exhibit the highest magnitude as follows:

(9) $$\left[\hat{m},\hat{k}\right]=\arg\underset{m,k}{\max}\left|Y_{m}\left[k\right]\right|,$$

where $\hat{m}$ is the estimated bin index, $\hat{k}$ is the estimated frequency index, and the symbol || denotes the absolute value function. Considering that the normal number of breaths per minute for an adult is about 12–20, the target respiration rate is limited to between 5 and 30 respirations per minute (RPM). Thus, frequencies below 0.083 Hz (5/60) or above 0.5 Hz (30/50) are filtered out. As shown in Figure 3, the maximum magnitude in the FFT output appears at 0.3125 Hz. In that experiment, the true value of breath frequency is also 0.3125 Hz (10/32). In this case, the estimated value is the same as the true value. In contrast, the frames received and the FFT output for non-stationary conditions are shown in Figure 4 and Figure 5, respectively.

In Figure 4, the received signal is distorted by the random shaking of the subject. As shown in Figure 5, the obtained frequency with maximum magnitude from the FFT output is 0.125 Hz. However, the true value of breathing frequency is about 0.34375 Hz (11/32). Obviously, the estimation error is large while the subject is non-stationary.

Hu and Qiu [31] point out that acceleration can simplify the characteristic changes of different micro-movements more than speed. In [34], the acceleration-based method [31] is used to select the received signal with minimal body movement. The acceleration factor can be expressed as

(10) $$a[n,m]=\frac{4y[n,m]+\left(y[n+1,m]+y[n-1,m]\right)-2\left(y[n+2,m]+y[n-2,m]\right)-\left(y[n+3,m]+y[n-3,m]\right)}{16T^{2}}.$$

**Figure 6:**
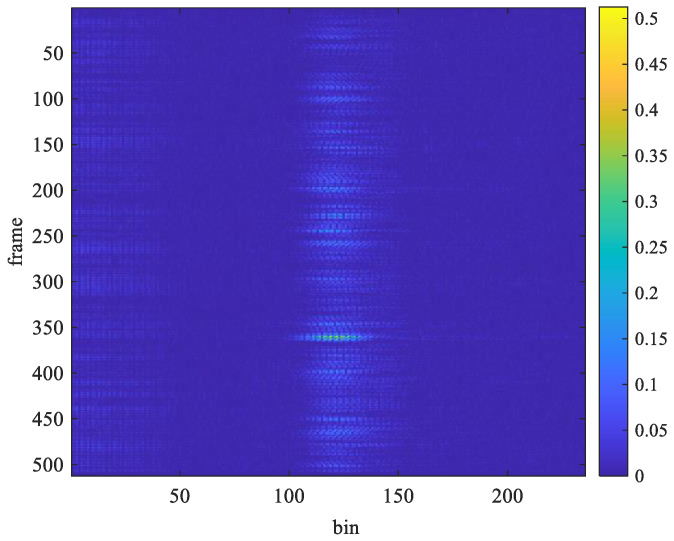
The acceleration factor values when the subject is stationary.

**Figure 7:**
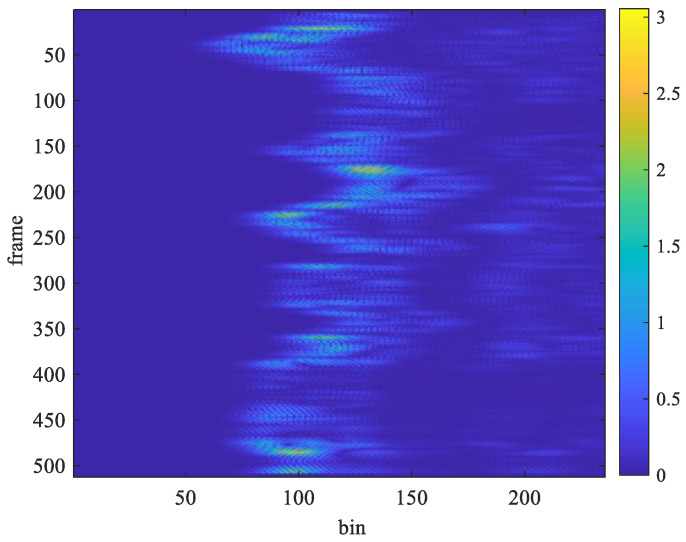
The acceleration factor values when the subject moves randomly.

The acceleration factor of all received signals is calculated through Equation (10), as displayed in Figure 6 and Figure 7. The acceleration factor of the respiration signal measured in the stationary state is small, as shown in Figure 6. When the subject makes some random movements during the measurement process, a large acceleration will be observed, as shown in Figure 7. Thus, the acceleration factor can be used to determine whether the subject was stationary during measurement and can help filter out receiving signals to improve the accuracy of measurement.

In [34], if the acceleration factor exceeds a threshold, the received sample is marked as 1; otherwise, it is marked as 0. Then, the block with the smallest number of 1 s is selected. However, the threshold is difficult to determine because it is related to propagation attenuation and the radar cross section (RCS) of the human body. Therefore, directly comparing the acceleration factors at the same bin is proposed to avoid the problem of selecting a threshold. As mentioned above, each bin contains *N* acceleration factors. In this article, we propose to divide the *N* acceleration factors into *K* overlapping windows. The number of windows for each bin can be computed by

(11) $$K=\left\lfloor \frac{N-W}{W-L}\right\rfloor +1,$$

where *N* is total number of frames, *W* is window length, *L* is overlapping length, and the ⌊ ⌋ symbol denotes the floor function. For example, the window length was set to 256 frames, and the overlap was 128 frames. Thus, a 512-frame block was divided into 3 overlapping windows.

Then, the acceleration factors in each window were summed, and the window with the smallest sum was selected. The total number of the acceleration factors in the *i*-th window and the *m*-th bin is given by

(12) $$R\left[v,\;m\right]=\sum_{n=\left(v-1\right)W+1}^{vW}a\left[n,m\right],$$

where v satisfies 1≤v≤K and *m* satisfies 1≤m≤M. Then, for each bin, the window with the smallest total number was selected as follows:

(13) $${\hat{v}}_{m}=\arg\underset{1\leq v\leq K}{\min}R\left[v,m\right].$$

where ${\hat{v}}_{m}$ satisfies $1\leq {\hat{v}}_{m}\leq K$. Thus, the selected sample matrix can be expressed as

(14) $$\hat{y}=\left[\begin{array}{ccc} y\left[\left({\hat{v}}_{1}-1\right)W+1,1\right] & \cdots & y\left[\left({\hat{v}}_{M}-1\right)W+1,M\right]\\ \vdots & \ddots & \vdots \\ y\left[{\hat{v}}_{1}W,1\right] & \cdots & y\left[{\hat{v}}_{M}W,M\right] \end{array}\right].$$

Finally, a 256-point FFT was performed on the selected sample matrix, $\hat{y}$, and the respiratory frequency was determined by selecting the frequency with the highest magnitude. The FFT output for *m*-th column (bin) of the selected sample matrix can be computed by

(15) $${\hat{Y}}_{m}\left[k\right]=\sum_{i=1}^{W}\hat{y}[i,m]e^{-j2\pi k(i-1)/N}.$$

**Figure 8:**
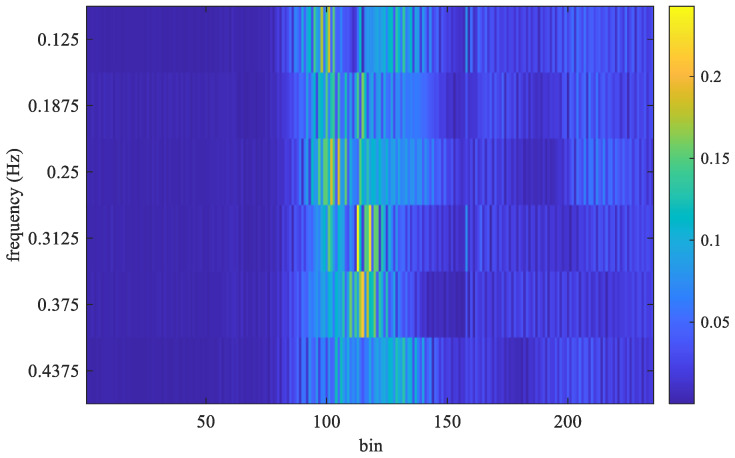
FFT output of the selected window.

As shown in Figure 8, the highest magnitude appears at the frequency of 0.3125 Hz, but the true value is 0.34375 Hz. Because the window length is 256, the FFT frequency resolution is 0.0625 Hz (16/256). It is worth noting that the difference between the estimated value and the true value, i.e., the estimation error, is also caused by the limitation of the FFT frequency resolution.

**Figure 9:**
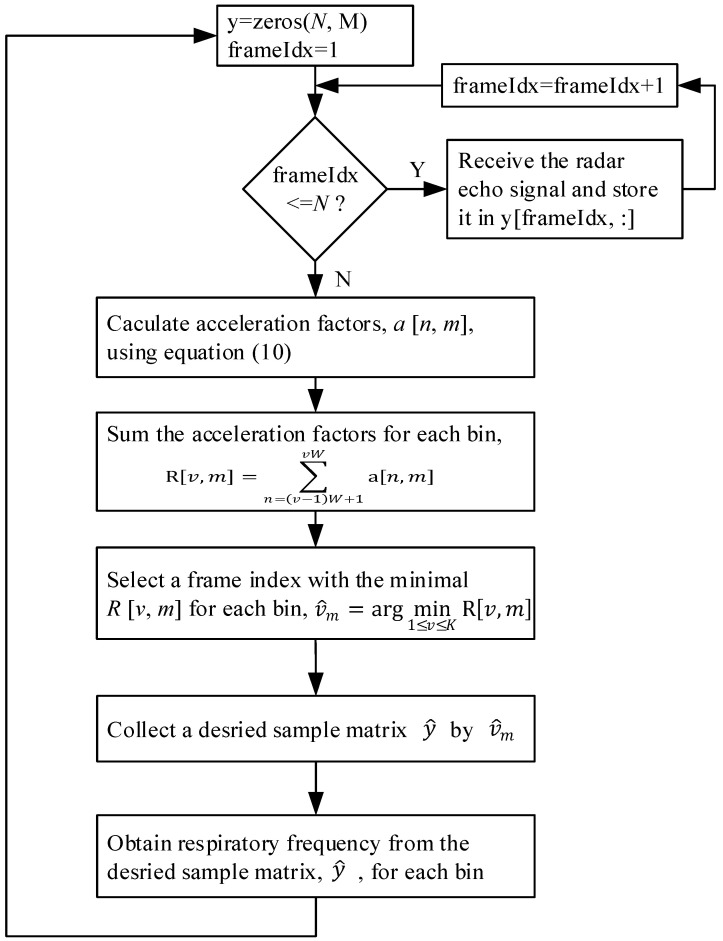
Flowchart for the proposed adaptive least motion window selection (ALMWS) algorithm.

The flowchart in Figure 9 illustrates the proposed adaptive least motion window selection (ALMWS) algorithm. First, *N* frames of radar echo signals are received and stored in array y, with each frame containing *M* samples. Once the *N* frames are collected, the acceleration factor for each sample is calculated. Next, for the samples in each column (bin), a window of size *W* is applied to calculate the sum of the acceleration factor values for the samples within the window, as described in Equation (12). The window with the smallest sum of acceleration factor values is selected in each column, as described in Equation (13). The samples within this selected window are then stored in the array $\hat{y}$. Finally, the respiration frequency is extracted from the array $\hat{y}$ using the FFT, as described in Equations (8) and (9).

The window length *W* in Equation (11) significantly impacts the estimation performance. It involves a trade-off between time and frequency resolution. A shorter window provides better time resolution but lower frequency resolution. In contrast, a longer window enhances frequency resolution but reduces time resolution, making it more suitable for stationary signals. The optimal choice depends on the characteristics of the signal. For instance, when a subject is stationary, a larger window length should be used to reduce estimation errors. However, when the subject is in motion, a smaller window length should be chosen to minimize interference from movement-induced signals. Especially when the subject is moving, the distance between the body and the UWB radar changes, leading to discontinuities in the sampled signal along the slow-time axis, as shown in Figure 7. In this paper, the window length is determined based on the subject’s motion status. When the subject is stationary, only the chest movement caused by breathing occurs, resulting in a smaller acceleration factor, as shown in Figure 6. When the subject is non-stationary, such as walking back and forth, a significantly larger acceleration factor is generated, as shown in Figure 7. If only the subject moves in the environment while all other objects remain stationary, all unwanted clutters will be removed, and the subject’s location can be determined using the acceleration factor in both Figure 6 and Figure 7.

Selecting a signal block from the acceleration factor matrix a, which contains $N_{f}$ frames and $T_{w}$ bins, the signal block is given by

(16) $$A_{n,m}=\left[\begin{array}{ccc} a[n,m] & \cdots & a[n,m+T_{w}-1]\\ \vdots & \ddots & \vdots \\ a[n+N_{f}-1,m] & \cdots & a[n+N_{f}-1,m+T_{w}-1] \end{array}\right],$$

where $1\leq n\leq {N-N}_{f}+1$ and $1\leq m\leq M-T_{w}+1$. As each row of the signal block is periodically sampled with a time interval of $T_{f}$, the signals in each column are summed to enhance the signal-to-noise ratio and then squared, following the method in [35], as shown in the following equation:

(17) $$z\left[n,m\right]=\sum_{i=0}^{T_{w-1}}{\left(\sum_{j=0}^{N_{f}-1}a[n+j,m+i]\right)}^{2},\;$$

For each slow-time index *n*, the distance between the UWB radar and the subject, measured in the number of bins, can be estimated by identifying the value of *m* that maximizes $z\left[n,m\right]$, as shown in the following equation:

(18) $$d\left[n\right]=\underset{m}{\mathrm{arg max}}z\left[n,m\right].$$

Although there is a fixed offset between *d*[*n*] in Equation (18) and the bin where the subject is located, the subject’s movement can still be determined by observing whether *d*[*n*] continues to change over time. If the value of *d*[*n*] fluctuates beyond a threshold, denoted as γ, and persists for a certain duration τ, such as 1 s, it is marked as movement. Among N frames, the longest segment that does not contain any movement marks is identified, and its length is denoted as λ. The window length is then set to the largest power of 2 that is closest to but does not exceed λ. Additionally, to prevent excessive estimation errors caused by low frequency resolution when the window length is too small, a minimum window length can be defined, such as 64. The algorithm is described as follows.

⟡ Inputs:
  - *N:* The maximum window length.
  - *F_s_*: Frame rate (frames per second).
  - *γ*: A threshold value (in bins) for the movement distance.
  - τ: The continuous time (in seconds) for which the subject must exceed the movement threshold to consider a significant movement.
  - *d*: An array of distances between the UWB radar and the subject. This is a 1 by *N* array
⟡ Output:
  - *W*: The target window length.
⟡ Variables:
  - *i*: The frame index, used to iterate through the array *d*, which contains the distances at each frame.
  - *lastMovedFrame*: Tracks of the last frame index where significant movement was detected. It is used to determine the frame where movement stops (i.e., the frame where the subject stops moving significantly).
  - *ExectedThresholdCnt*: A counter that tracks how many consecutive frames have exceeded the threshold distance γ, indicating that the subject has been moving continuously over that threshold.
  - *Interval*: This is a 1 by N array. When a frame is marked as moving, this array records the number of frames between the last moving frame and the current moving frame.
⟡ Process:
  - The loop processes each frame, comparing the distance *d*[*i*] with the distance *d*[*lastMovedFrame*].
  - If the distance change (in bins) is greater than the threshold γ, the counter *ExectedThresholdCnt* is incremented.
  - If *ExectedThresholdCnt* exceeds *F_s_* * τ (i.e., the subject has been moving continuously for time τ), the interval from the *lastMovedFrame* to the current frame (*i* − *lastMovedFrame* + 1) is stored in *Interval* array, and the frame index is set as the new *lastMovedFrame*.
  - If the change in distance is less than γ, the counter *ExectedThresholdCnt* is reset.
  - Finally, the maximum value in *interval* array, denoted as λ, is found, and *W* is set to the largest power of 2 less than or equal to λ, with a lower bound of 64.

## 4. Experimental Results

**Figure 10:**
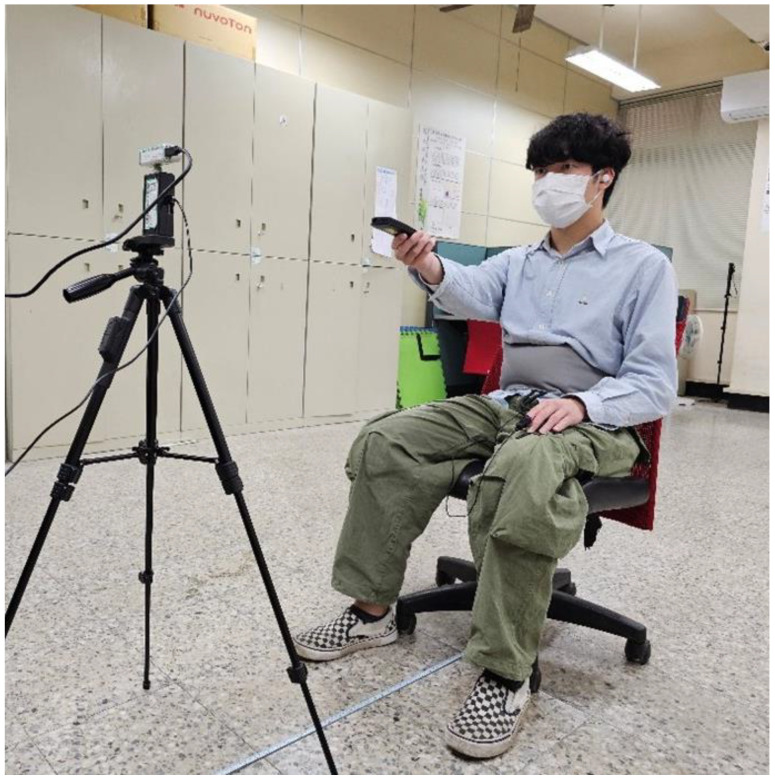
The experimental setup.

**Figure 11:**
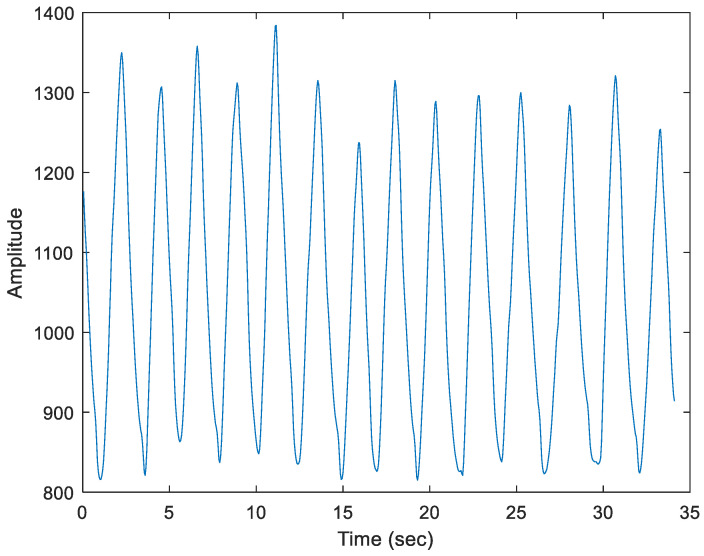
The signal recorded from the NeuLog NUL-236 respiratory belt.

The experimental setup is illustrated in Figure 10. The measurement range is between 0.5 and 2 m, conducted in a low-interference environment. The distance between the subject and the X4M200 is set to 1 m. The subject wears a NeuLog NUL-236 respiratory belt (Scientific Educational Systems Ltd., Rishon Lezion, Israel), and the recorded signal is shown in Figure 11. The data detected by the respiratory belt sensor are transmitted to the receiving computer through the NeuLog RF-201 wireless transceiver module (Scientific Educational Systems Ltd., Rishon Lezion, Israel). Simultaneously, the RGB images and depth maps captured by the RealSense D435i (Intel Corporation, Santa Clara, CA, USA), along with the radar echoes received by the X4M200 (Novelda, Oslo, Norway), are transmitted to the computer. The MATLAB (R2024a) program used for data collection stores the respiratory signals, UWB radar echoes, RGB images, and depth maps in a single MATLAB data file (.mat file) for subsequent performance evaluation.

As shown in Figure 11, the breathing rate is generally stable over a short period. Therefore, estimating the breathing rate using a segment of radar echo signals will not introduce significant errors. Moreover, the error rate in this case is lower than that of estimating the breathing rate from radar echo signals when the body is in motion.

**Figure 12:**
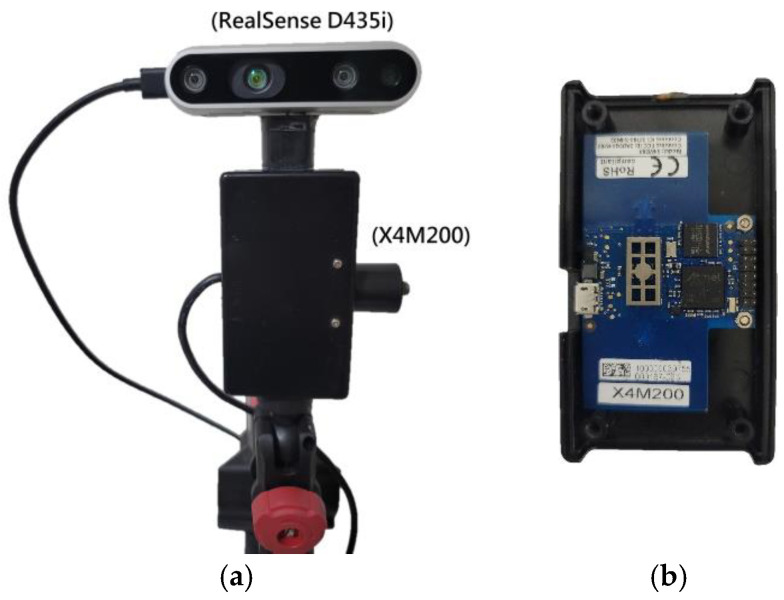
The experimental setup includes the X4M200 UWB radar and RealSense camera. (**a**) RealSense D435i positioned directly above the X4M200, (**b**) X4M200 UWB radar module.

With the assistance of image recognition technology, cameras can effectively identify key points in the human body, making it possible to determine whether the body is in motion. In this paper, we also compare the performance of the proposed algorithm with a fusion of UWB radar and an RGB-D camera-assisted scheme [36,37]. This experiment utilizes an RealSense RGB-D camera. As shown in Figure 12, the RGB-D camera is positioned directly above the X4M200 UWB radar. The RGB-D camera is used to detect whether the subject is moving.

**Figure 13:**
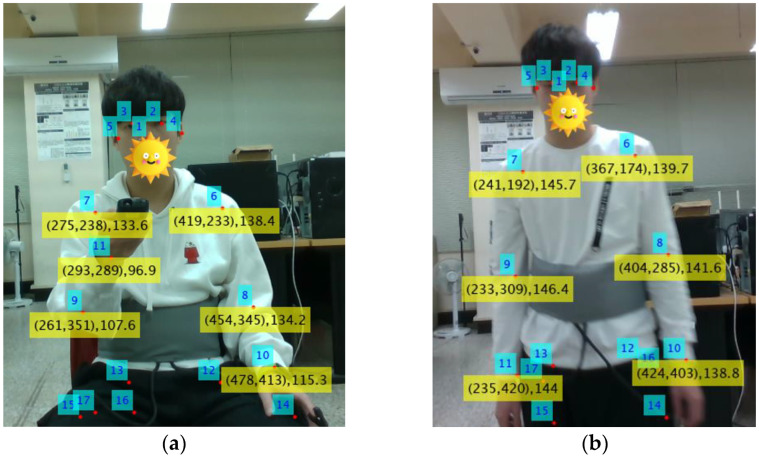
RGB images and distance information were obtained through RealSense. (**a**) The subject is sitting on a chair and operating a remote control. (**b**) The subject is walking back and forth.

RGB images and depth information can be obtained using the RealSense camera. Leveraging image recognition technology, the pixel coordinates of key points on the human body can be identified from RGB images. In Figure 13, the subject’s key points are highlighted from an image captured by RealSense. Using the pixel coordinates of these key points, the corresponding distances can be determined from the depth image. For instance, in Figure 13a, the pixel coordinates of key point number 6 (left shoulder) are (419, 233), and its distance from RealSense is 138.4 cm.

In this experiment, six key points, numbered 6 (left shoulder), 7 (right shoulder), 8 (left elbow), 9 (right elbow), 10 (left wrist), and 11 (right wrist), are monitored to determine whether the subject is moving. Monitoring hand key points is particularly important because hand movements can interfere with accurate breathing detection. Unlike the method proposed in this paper, which uses Equation (10) to calculate the acceleration factor, the RGB-D camera-assisted method determines motion by analyzing changes in the distances of these key points.

Assuming the estimated frequency is $\hat{f_{b}}$, and the estimation error per minute, ε, is defined as $\varepsilon =\left|f_{b}-\hat{f_{b}}\right|\times 60$. The RMSE performance is measured by the root mean square of ε. Thus, the RMSE is given by

(19) $$RMSE=\sqrt{E\{\varepsilon^{2}\}},$$

where $E\{\varepsilon^{2}\}$ denotes the mean value of the square of the estimation error. In Table 2, the experimental results of the proposed scheme are compared with those of the FFT, STFT and RGB-D camera-assisted schemes. In this experiment, the STFT scheme uses the Kaiser window. The experimental conditions are illustrated in Figure 10. The frame rate is set to 30 Hz (i.e., $T_{f}=1/30$ s) and *N* is set to 1024. The FFT method simply performs the FFT on the received 1024 × 235 sample matrix and determines the respiration frequency by selecting the frequency with the highest magnitude, as shown in Equation (9). In our experiments, the window length (*W*) is adaptively selected, and the overlap is equal to *W*-1 frames for the proposed scheme, the STFT, and the RGB-D camera-assisted schemes. Please note that the proposed scheme only performs a single *W*-point FFT for the samples in the selected window, but the STFT scheme must perform a separate *W*-point FFT for samples in each candidate window. Therefore, the computational complexity of the proposed scheme is much lower than that of the STFT scheme. In Table 2, the RMSE performance of the FFT, the proposed algorithm, the RGB-D camera-assisted, and the STFT schemes is compared. The test cases include:

1. Stationary
2. Operating a remote control for 5 s, then stopping for 5 s
3. Operating a remote control for 10 s, then stopping for 10 s
4. Operating a remote control for 15 s, then stopping for 15 s
5. Walking back and forth for 5 s, then stopping for 5 s
6. Walking back and forth for 10 s, then stopping for 10 s
7. Walking back and forth for 15 s, then stopping for 15 s

For Test Cases 2, 3 and 4, the subject sat in a chair while operating the remote control, as shown in Figure 13a. For Test Cases 5, 6 and 7, the subject walked back and forth, as illustrated in Figure 13b.

**Figure 14:**
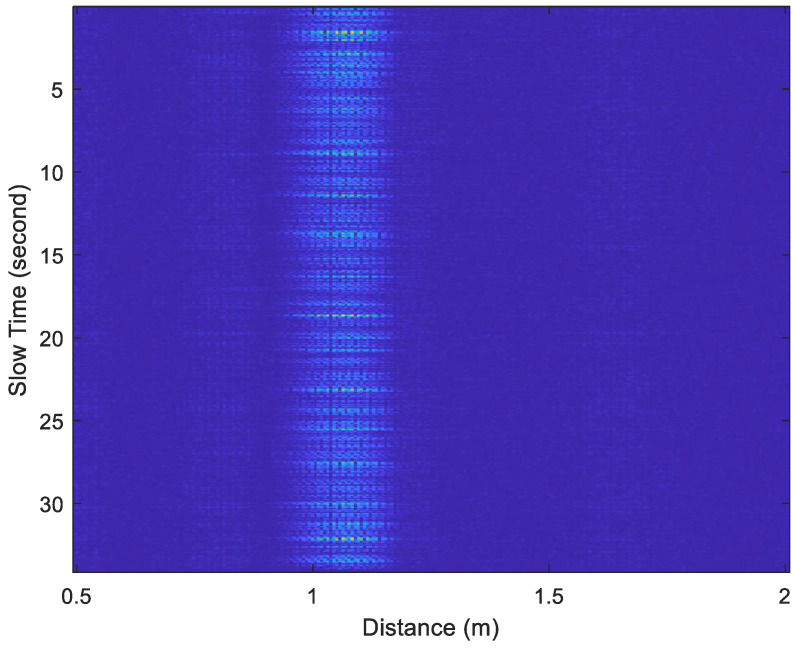
The acceleration factor of radar echo signals recorded under Test Case 1.

**Figure 15:**
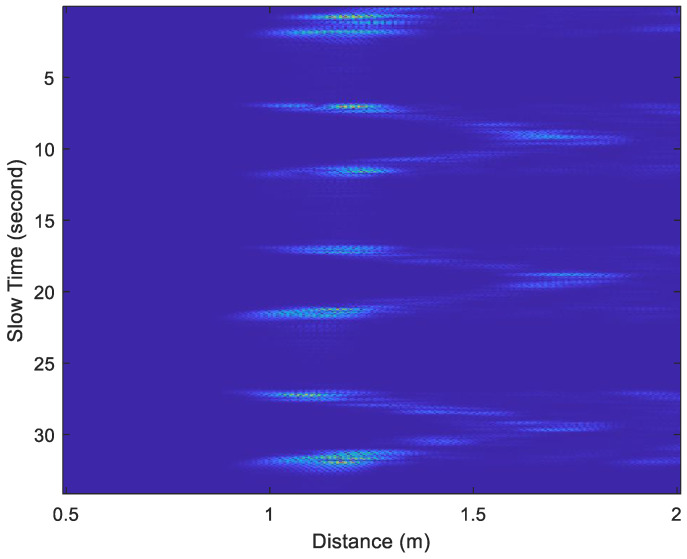
The acceleration factor of radar echo signals recorded under Test Case 5.

**Figure 16:**
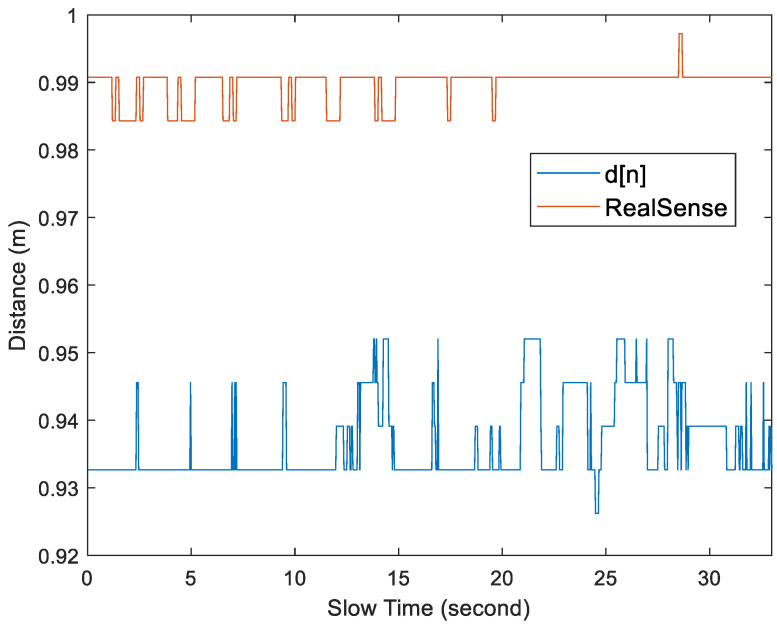
Plot of the distance between the subject and the UWB radar under Test Case 1.

**Figure 17:**
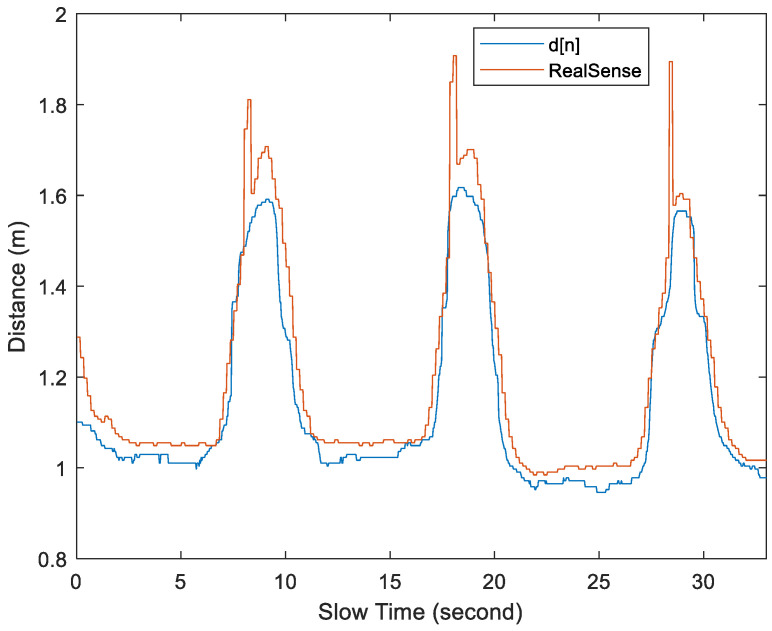
Plot of the distance between the subject and the UWB radar under Test Case 5.

Figure 14 and Figure 15 show examples of the acceleration factor of radar echo signals recorded under Test Case 1 and Test Case 5, respectively. The slow-time axis represents the frame index divided by 30. By substituting the data from Figure 14 and Figure 15 into Equations (17) and (18), with $T_{w}$ set to 40 bins, the *d*[*n*] plots in Figure 16 and Figure 17 were obtained, respectively.

In Figure 16 and Figure 17, when measuring the distance with RealSense, the average depth of field measured by key points 6 and 7 was used. By comparing the distances measured by RealSense and *d*[*n*] in Figure 16 and Figure 17, a displacement is observed between the two, but the relative trends remain consistent. When the subject is stationary, the values measured by RealSense are more accurate. However, when the subject is in motion, there is a larger error.

Table 2 details the RMSE performance per minute. When the subject is stationary, such as in Test Case 1, the RMSE performance depends on the frequency resolution. If the window length is 1024, the frequency resolution is 30/1024 = 0.029 Hz. Therefore, the resolution is 60 × (30/1024) = 1.76 breaths per minute (BPM). This resolution (1.76 BPM) defines the potential range of errors due to frequency quantization. Assuming a uniform distribution of error between 0 and 1.76 BPM, the average error is 0.88 BPM. This represents the lower bound of error under stationary condition, with the frame rate set to 30 Hz and the window length set to 1024. In Test Case 1, the FFT scheme utilizes all 1024 frames, while the other schemes use only a subset of the frames. As a result, the FFT and RGB-D camera-assist methods perform better than the other methods because the subject is stationary, allowing the complete 1024 frames of samples to be used for estimation. In contrast, the proposed method and the STFT method sometimes use a window length smaller than 1024.

In Test Cases 2 to 7, where the subjects are non-stationary, the FFT method performs relatively poorly. This is due to interference from operating the remote control or time shifts caused by walking back and forth, which affect some of the received echo signals. In contrast, the STFT, RGB-D camera assist, and the proposed method in the table use adaptive window length to reduce interference caused by the subject’s movement. The parameters were set to *γ* = 4 bins (i.e., 4 × 0.65 = 2.6 cm), $T_{w}\;$ = 40 bins and τ = 1 s. To fairly compare the difference in performance between our proposed method and STFT, the same window length was used for STFT as in our proposed scheme. The STFT method performs better than FFT because it selects the frequency with the largest amplitude from the sliding time-frequency window. This approach helps avoid time periods that may be affected by human motion, potentially leading to more accurate results than the FFT scheme. However, it still performs worse than the proposed scheme and the RGB-D camera-assisted scheme. This is because the STFT scheme directly selects the frequency with the largest amplitude from the sliding time-frequency window, whereas the proposed algorithm and the RGB-D camera-assisted scheme select the window with the least movement, which helps to minimize interference from body motion and improve accuracy.

**Figure 18:**
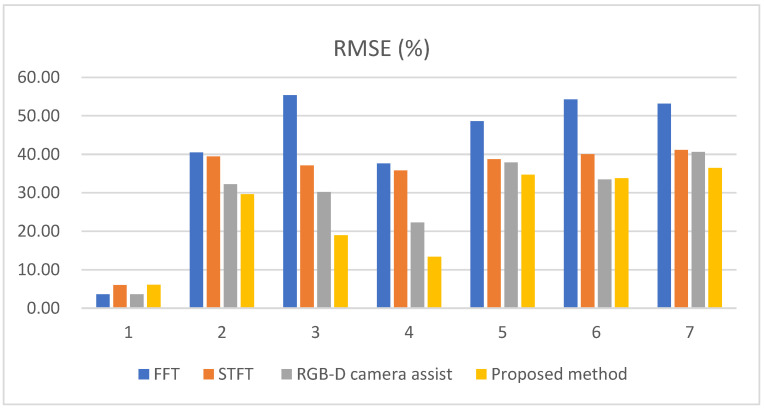
The percentage of the number of errors per minute in Table 2, divided by the true average value.

The proposed scheme outperforms the RGB-D camera-assisted scheme in Test Cases 2 to 4 and slightly outperforms it in Test cases 5 to 7. According to the data sheet, the depth accuracy of the D435i camera at a 2 m working distance is less than 2%. This means there could be an error between 2 and 4 cm at distances ranging from 1 to 2 m. As shown in Figure 16, with the *γ* = 4 bins set, the stationary subjects were almost never misclassified as moving. However, as shown in Figure 17, when the subject is in a moving state, RealSense D435i will experience more significant errors, potentially leading to some misjudgments. Figure 18 shows the percentage of the number of errors per minute in Table 2, divided by the true average value. It is clearly evident that the method we propose demonstrates better performance.

## 5. Conclusions

In this paper, an algorithm has been proposed to effectively detect the respiratory rate of a non-stationary individual. The main contribution of this paper is the development of an algorithm to determine appropriate window length and select the minimum number of samples with the least movement. By adaptively determining a suitable window length and calculating the acceleration factor, the window block with the smallest accumulated acceleration factors was selected. Experimental results demonstrate that the proposed scheme outperforms the FFT, STFT, and RGB-D camera-assisted methods under non-stationary conditions. Moreover, the computational complexity of the proposed scheme is lower than that of both the STFT and RGB-D camera-assisted methods, confirming its effectiveness. Additionally, Test Cases 5, 6, and 7 exhibit large RMSE errors. In this paper, we have proposed a method for measuring the distance between the UWB radar and the subject. This distance measurement technique can be used to synchronize radar echo signals reflected from the subject. Future work will focus on how to merge phase-discontinuous signals to extract more complete respiratory signals.

## Figures and Tables

**Table 1:** Experimental conditions.

Item	Condition
**Antenna height from the ground**	0.95 m
**Carrier frequency**	7.29 GHz
**Sampling frequency (** $\frac{1}{T_{∆}}$ **)**	23.328 GHz
**Frame rate (** $\frac{1}{T_{f}}$ **)**	16

**Table 2:** RMSE performance comparison per minute (BPM).

Test Case	RMSE				True Average Value
	FFT	STFT	RGB-D Camera Assist	Proposed Method	
1	0.93	1.55	0.93	1.56	25.64
2	10.46	10.20	8.33	7.66	25.86
3	14.45	9.68	7.87	4.95	26.10
4	9.90	9.43	5.86	3.53	26.33
5	12.73	10.14	9.93	9.08	26.21
6	14.67	10.83	9.04	9.13	27.05
7	14.03	10.86	10.72	9.61	26.39

## Data Availability

Dataset available on request from the authors.
